# Dynamic Conservation in Risk Society: A Case Study of COVID-19 Pandemic Risk in Kashan Qanat Irrigated Agriculture

**DOI:** 10.3389/fpubh.2022.882943

**Published:** 2022-05-09

**Authors:** Mohammad ali Manian, Korous Khoshbakht, Hossein Mahmoudi, Houman Liaghati

**Affiliations:** Environmental Sciences Research Institute, Shahid Beheshti University, Tehran, Iran

**Keywords:** agro-biodiversity, traditional agriculture, GIAHS, COVID-19 pandemic, sustainable rural development, agroecology

## Abstract

In the present age, the world agricultural heritage can inspire agroecology and sustainable agriculture. But various risks have threatened, eroded and forgotten this heritage, so dynamic conservation of this heritage is essential. In this study, “Qanat Irrigated Agricultural Heritage Systems, Kashan, Iran” which has been registered worldwide in the face of corona pandemic risks has been selected as a case study. In this qualitative research, in addition to field observations and documentary studies, 25 in-depth interviews and 39 semi-structured interviews with experts and key informants was done and grounded theory and content analysis have been used. In the process of interviews and analyzes based on “risk society theory”, risks and wicked problems and related solutions have been identified and finally based on cultural theory, “clumsy solution space” has been summarized and presented for dynamic conservation. Based on the findings of this study, paying attention to a kind of reward for ecosystem services, developing online sales of agricultural products in rural areas of Kashan and also creating twinning with similar areas can help solve wicked problems. Also, paying more attention to the regulations for the protection of qanats, as well as the laws for home business insurance, can strengthen sustainable development in this rural area. Due to the wide range of different dimensions of agricultural heritage, it is suggested that in future research, clumsy solution spaces for each of these dimensions be created and developed separately.

## Introduction

The persistence of agricultural heritage systems reflects the ability and ingenuity of farmers who have passed on their experiences from generation to generation in order to adapt to unpredictable environmental changes (1). It should be noted that such environmental and cultural treasures are of fundamental value for the future of mankind. The guardians of these systems show a lasting commitment to the protection and respect of nature, ancestral agriculture and its valuable treasures, although modern agriculture continues to threaten sustainability and heritage.

Due to the special climatic conditions and history of agricultural civilization, Iran has many examples of agricultural heritage. In the introduction to the Persian translation of the book Agroecology, Gliessman wrote that traditional Iranian farmers are famous for their ability to design and manage sustainable agroecosystems and are among the first agroecologists. The vast knowledge of these people in the protection of water resources, agriculture in arid areas, irrigation systems, domestication of crops and livestock, which are just examples of their unlimited capabilities, have been used for years as models of wise management of natural resources in restrictive conditions (2). So far, three examples of systems related to Iran's agricultural heritage have been registered globally in the important global agricultural heritage systems (GIAHS). Iran has registered the “Qanat Irrigated Agricultural Heritage Systems, Kashan” in 2014, the “Qanat-based Saffron Farming System in Gonabad” and the “Grape Production System in Jowzan Valley” in 2018, and the other three systems Estahbanat Rainfed Fig Orchards Heritage System, Ancient Traditional Gardens of Qazvin and Traditional Walnut Production Systems in Tuyserkan also Proposed. None of Iran's agriculture heritage systems has yet had a comprehensive and effective action plan for dynamic conservation, and various components of these systems, including indigenous knowledge and prevented the formation of dynamic conservation of agricultural heritage in recent years is the existence of various and emerging hazards, which are addressed in this article with approaches related to the theory of risk society. However, after the advent of the COVID-19, global attention turned again to the theory of risk society (3). At the same time, the effects of the Corona pandemic in the fields of agriculture and agroecology were considered (4, 5). In this study, in addition to a general study of the impact of risk community parameters on agricultural heritage, a special look has been taken at the dangerous effects of COVID-19.

### Agricultural Heritage

Traditional agriculture in diverse environments has been created by indigenous peoples and local communities and has been shaped by the dynamic interaction of people and nature over time. These landscapes are rich in biodiversity, agricultural biodiversity, cultural and moral values and embody human genius (6, 7). Agricultural heritage systems produce food and human needs and provide their livelihoods locally and nationally, and are intelligent agroecological systems that benefit from the management and conservation of biodiversity and soil and water resources, and provide a variety of ecosystem services. Agricultural heritage systems and landscapes represent the human heritage on the planet and are evidence of the maturity, creativity and innovation of human beings in the dynamism and evolution of agroecological methods and the sustainable management and exploitation of nature. Ensuring sustainable food and livelihoods for indigenous and local communities, preserving natural ecosystems and basic resources, protecting animal and plant biodiversity, increasing the resilience of human communities to climate and environmental stresses, educating on issues related to sustainability and resilience to global change And the creation of visual beauty can be considered as one of the most important functions of agricultural landscapes and its ancient and sustainable systems (6, 8). Agricultural heritage patterns are an evolved, multilayered concept that connects indigenous and local communities, the environment, culture, history, and indigenous knowledge, and depicts the interaction between man and nature in a natural context. Agricultural heritage landscapes and traditional systems around the world are rapidly deteriorating due to modernization and changes resulting from unsustainable technologies and economic methods. Currently, the tangible and intangible values of agricultural heritage are identified and registered by UNESCO as Cultural Heritage Landscapes and the World Heritage List (GIAHS), and various conservation programs are proposed to protect them.

According to the FAO (2002) definition, the world's most important agricultural heritage systems (GIAHS) are “Remarkable land use systems and landscapes which are rich in globally significant biological diversity evolving from the co-adaptation of a community with its environment and its needs and aspirations for sustainable development”.

### Dynamic Conservation

Important global agricultural heritage systems are representations of the many traditional sustainable agricultural systems that have historically spread throughout the world and are preserved and protected by indigenous and local communities. The persistence of these systems in successive eras reflects the ability and ingenuity of farmers who have used a variety of strategies to adapt to environmental and climatic constraints and their unpredictable changes and have passed on their experiences from generation to generation (1) and Have left treasure of valuable culture for the future in this process; Thus, examples of GIAHS are considered as the centers of life and growth of agricultural heritage and are an inspiring source for sustainable agriculture in the new era. Since the richness of knowledge and experience related to sustainable resource management is evident in many important global agricultural heritage systems, it is essential to consider the protection and care of these systems as national and global treasures, while providing ample opportunity for growth and provide their dynamics (9, 10). In practice, the concept of—ample opportunity for growth and dynamism- inspires the patterns of dynamic conservation that are being designed today for each instance of GIAHS.

The concept of dynamic conservation is rooted in the idea that conservation is achieved not only in monuments, museums and untouched forests, but also in the daily lives of indigenous communities and according to their needs and expectations. GIAHS are a living and dynamic system that evolves over time, and absolute protection and prevention of any change and dynamism in these systems will reduce their capacity to adapt to new conditions, and this will lead to vulnerability and deterioration of agricultural heritage systems. In fact, over time, social, economic, and environmental needs change, creating a spirit of innovation and creativity, enabling individuals and communities to preserve traditional practices, products, and services and adapt them to new conditions. This process does not diminish the importance of agricultural heritage, and in this way, the fundamental values of historical systems and processes are created. For this reason, one of the pillars of strengthening GIAHS in each region is the capacity for innovative, product development, culturally identifiable services, handicrafts and local food, ecotourism, agricultural tourism, as well as a thriving market (9).

However, in many parts of the world, despite ongoing conservation efforts, many agricultural heritage sites and their associated ancient systems are affected by the negative consequences of modern agriculture, rapid urbanization, the quality of economic growth, and the weakening and degradation of basic agricultural resources. Water, soil, and biodiversity are at serious risk (11, 12). Such conditions further highlight the need for dynamic conservation of agricultural heritage systems.

Rich biodiversity as well as cultural diversity have historically been intertwined with agricultural heritage, and while they are considered the most important tourist attractions, they have inherently led to the dynamic conservation of these systems (13).

### Risky Society

The expansion of attention to society relations and risk is largely due to the theory of risk society proposed by the German sociologist Ulrich Beck. Despite his opposition, over time he found many like-minded people in various disciplines, including the environment (14). In most of his works, especially in his first work, Risk Society, which may be called his most influential book, Beck has argued that in the transition from industrial society, risk society is taking shape (15–21).

In the decade following the introduction of this theory, Beck and Giddens criticized, improved, expanded and integrated its various dimensions, and finally more development and explanation was achieved in the field of this theory, and some parameters were proposed about it (22, 23). The passage of time has provided numerous evidences and examples of this theory that are consistent with his theory. These examples and events began with the Chernobyl explosion, and today, with the advent of the COVID pandemic, the problems and turmoil created for human societies are being analyzed by some analysts based on the theory of risk society.

Although there are hypotheses about the role of humans in the emergence of the COVID-19 virus, if we ignore these hypotheses, the current human life style and lifestyle is such that it accelerates the progression of the virus, the amount of damage has increased and made human society vulnerable. This vulnerability has also paved the way for the application of risk society theory in these circumstances. In fact, phenomena such as the expansion of complex socio-economic communications through the most advanced means of transportation, high population density in metropolitan areas and massive trade in goods and materials around the world, which on the one hand shows human genius over other living things; It has caused a widespread and rapid spread of the virus among humans and has endangered the future of mankind. Corona is not limited to poor countries and has so far spread to more than 200 countries and its socio-economic consequences have affected almost all regions of the world; as the aftermath of phenomena such as the 2008 economic crisis and ISIS engulfed the entire world. Finally, it is interesting to note that among the warnings Beck gave more than two decades ago, in addition to referring to nuclear accidents and terrorist threats, he warned of a plague that would cause much more serious problems than we thought (17).

### Qanat Irrigated Agricultural Heritage Systems, Kashan

**Figure 1:**
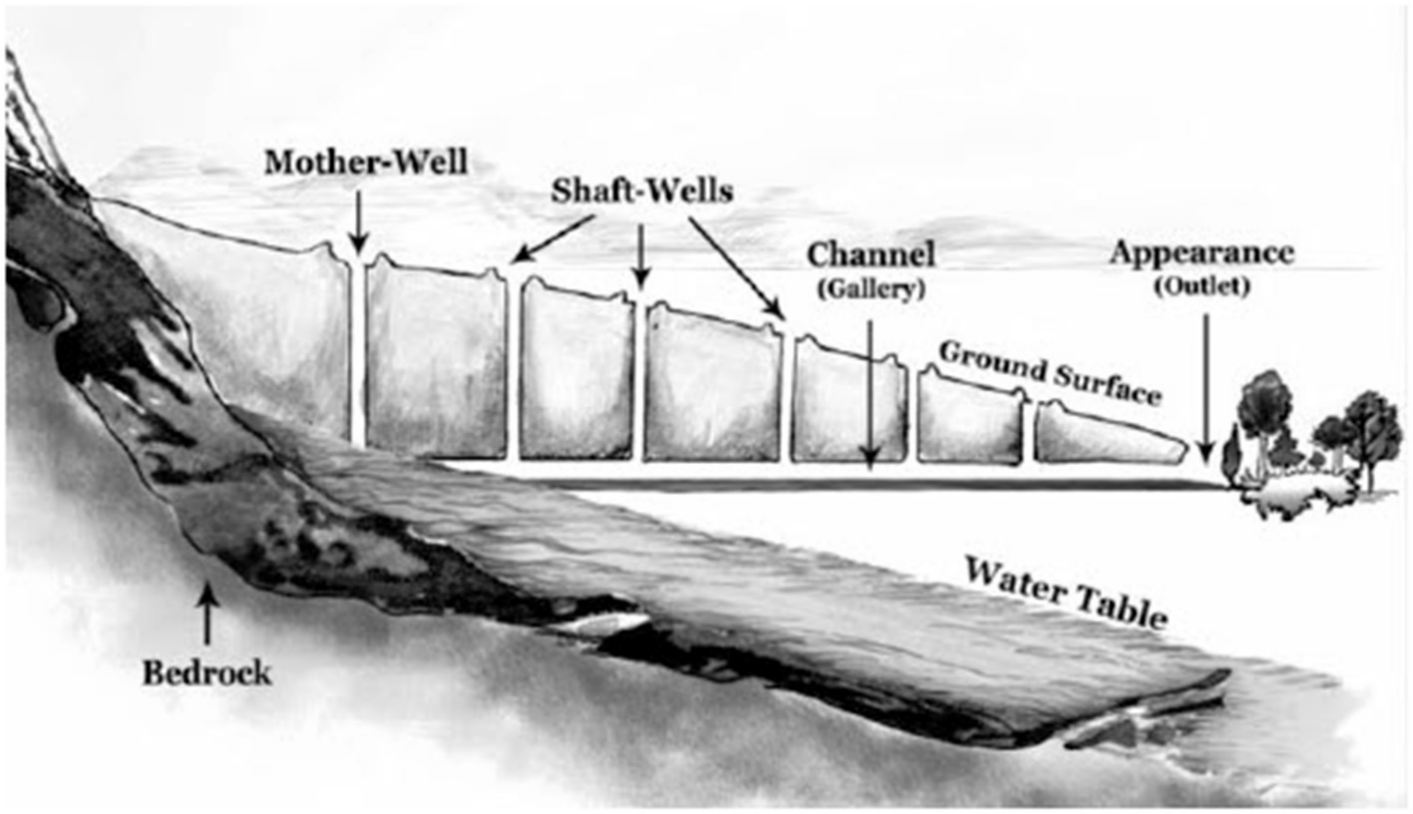
Profile of a typical qanat (24, 25).

An qanat as shown in Figure 1 is a collection of several wells and an underground water channel that, with a slope less than the slope of the earth's surface, the water in the layer (or layers) of the highlands of the earth with the help of gravity and Without using any extra energy, it collects with natural flow and brings it to lower points. In other words, the qanat can be considered as a kind of underground drainage through which the collected water is brought to the surface of the earth and is used for irrigation or drinking (23, 26, 27). This ancient technology was more prevalent in the Middle East, especially in Iran, and spread to North Africa, Spain, and South Asia. This method of water management can be considered as a sustainable method that can play a prominent role in water scarcity in the modern world (28, 29). There are more than 27 terms for qanats that are used in different countries: “Qanat” and “Kariz” in Iran, “Falaj” or “Aflaj” in Oman, “Kariz” or “Karez” in Afghanistan, Pakistan, Azerbaijan and Turkmenistan, “Ain” in Saudi Arabia, “Kahriz” in Iraq, “Kanerjing” in China, “Foggara” in Algeria, “Khattara” or “Khettara” and “Rhettara” in Morocco, “Galleria” in the Spain, “Qanat Romoni” in Syria and Jordan, “Foggara” and “Khettara” and “Iffeli” in North Africa, “Galerias” in the Canary Islands, “Mambo” in Japan, “Inguttati” in Sicily. Some other terms used for qanats are: Ghundat, Kona, Kunut, Kanat, Khad, Koniat, Khriga, Fokkara, etc. (30).

Numerous examples of the longevity and stability of the qanat are available. For example, according to some researches, Ghasabeh (Gonabad) qanat in Iran has a stable flow for about 2,500 years. In addition, according to the scientific interpretation today, the qanat is in line with the approaches of “smart agriculture to climate” and at the same time, with climate change, regulates its discharge so that resilience is maintained and the pressure is more than Environmental capacity should not be applied to the land where the qanat is located. Throughout the arid regions of Iran, water for permanent habitat and agriculture was supplied by the ancient qanat system from groundwater aquifers in valleys and the conduction of water along underground tunnels by gravity, often over miles. The traditional management system still enables the equitable and sustainable distribution and distribution of water in these areas. The qanat system provides exceptional evidence of cultural traditions and civilizations in arid desert areas. These features led the UNESCO World Heritage Committee to unanimously declare the 11 qanats of Iran a World Heritage Site.

The names of registered qanats, “Ghasabeh Gonabad, Baladeh Ferdows, Zarch Hassan abad Yazd, water mill Mirza Nasrollah Mehriz, Jupar Kerman, Akbar abad and ghasem abad Barvat Bam, Moon in Ardestan, Vazvan and Mozd abad of Isfahan and Ebrahim abad of Arak” in six provinces Razavi Khorasan, South Khorasan, Yazd, Kerman, Markazi and Isfahan are included and of course it should be mentioned that Kashan Fin Garden, which is irrigated through the Sulaymaniyah qanat spring, has also been registered by UNESCO (25). Water is supplied to a large number of Iranian gardens by qanats, and the risks that threaten qanats actually threaten Iranian gardens (26).

Despite the fact that qanats have historically formed very large oases, today they are under severe threat due to socio-economic pressures and technological changes. Of course, the special value of the qanat for the sustainable extraction of water has been considered by the United Nations and some other institutions, and they support and encourage the rehabilitation, protection and maintenance of the qanats in various ways (31).

**Figure 2:**
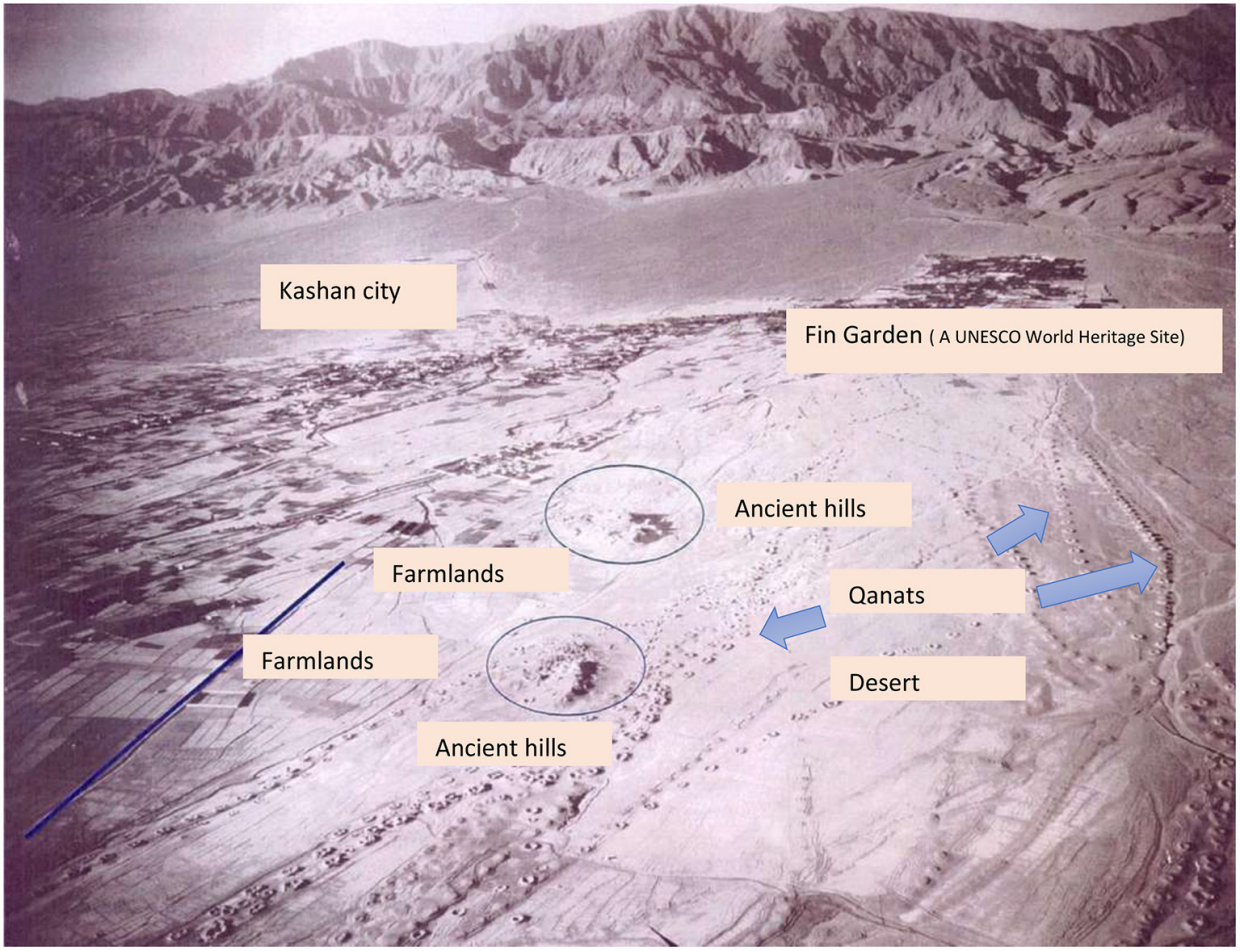
Aerial photograph of Kashan region around 1937 (25, 32).

One of the oldest qanat-based civilizations in central Iran as shown in Figure 2 is the 7,500-year-old Silk civilization, the remains of which are located in Kashan and near the Fin qanat (Sulaymaniyah spring), and research shows that the Fin qanat is the result of turning the spring into the qanat has been used by the inhabitants of this region throughout history, and of course the conversion of a spring into an qanat is one of the hypotheses about the emergence of the first qanats (24). This region was registered as an important world agricultural heritage in 2014, and the complete file of Kashan GIAHS under the title “Qanat Irrigated Agricultural Heritage Systems, Kashan, Iran” has been published by the FAO and provides valuable information about the features of global interest (25).

Throughout history, the genius and empowerment of the local community has used a variety of tools to deal with risks such as water scarcity, and carpet weaving is a prime example of this. Kashan carpet has a world-wide reputation. Carpet weaving has been one of the pillars of Kashan GIAHS resilience and prosperity. Throughout history, whenever the qanats of Kashan faced water shortages and naturally the activity and income of farming decreased, the focus of the people of this GIAHS on the art of carpet weaving increased. In fact, carpet weaving was done with raw materials that were available from previous years or purchased from other regions, and to some extent, the reduction of agricultural income was compensated. In addition, the cost of maintaining the qanat has been high in some areas, and the income from carpet weaving has also helped maintain the qanat. Especially at times when the qanat was damaged due to natural disasters such as floods and earthquakes, and its repair required higher than usual costs. In the Corona pandemic, carpet weaving has flourished at home due to the increase in housekeeping and the decrease in many incomes in this area. Of course, the purchase of carpets has decreased and problems such as carpet weavers' insurance are raised, but in general, carpet weaving has also played its resilient role in this period of the history of this GIAHS.

At the end of the introduction and after introducing the basic and introductory concepts, it is necessary to emphasize: Based on the literature review, it was found that many studies on dynamic conservation of agricultural heritage in rural communities have identified and explained their components and relationships. In fact, the need to address the concept of risk became apparent. In other words, there is a gap in addressing the concept of risk in the field of dynamic conservation against wicked problems, and in this research, an attempt is made to fill this gap to some extent.

Also, the obvious innovation of this research is in applying the “risk society” approach in dynamic conservation in the face of complex problems. On the other hand, given the emerging risks associated with the Corona pandemic that threaten sustainable rural development, it is necessary to pay attention to such new risks in such areas and has an innovative aspect.

The question of this research is what are the solutions to face the risks and wicked problems in Kashan GIAHS as a predominantly rural area during the Corona pandemic? Some of these problems generally hinder sustainable development in the region, while others are directly related to COVID-19. In order to better understand the problems and their solutions, it is necessary to understand the different dimensions of agricultural heritage and also to identify the relationship between the risk society, dynamic conservation and GIAHS, which have also been addressed.

To achieve the goals of this study, it is necessary to know, study and examine the situation of a rural community as a case study. This area should be a rural area with valuable agricultural heritage. Such an area needs to be relatively stable throughout history and to be exposed to new risks in the present era. According to the definition provided by GIAHS, such areas have the mentioned characteristics, it is enough that this area is in a significant position in terms of exposure to corona-related risks. According to the explanations provided in the sections “Qanat Irrigated Agricultural Heritage Systems,” and “study area” Kashan GIAHS was selected for this purpose.

## Materials and Methods

### Study Area

**Figure 3:**
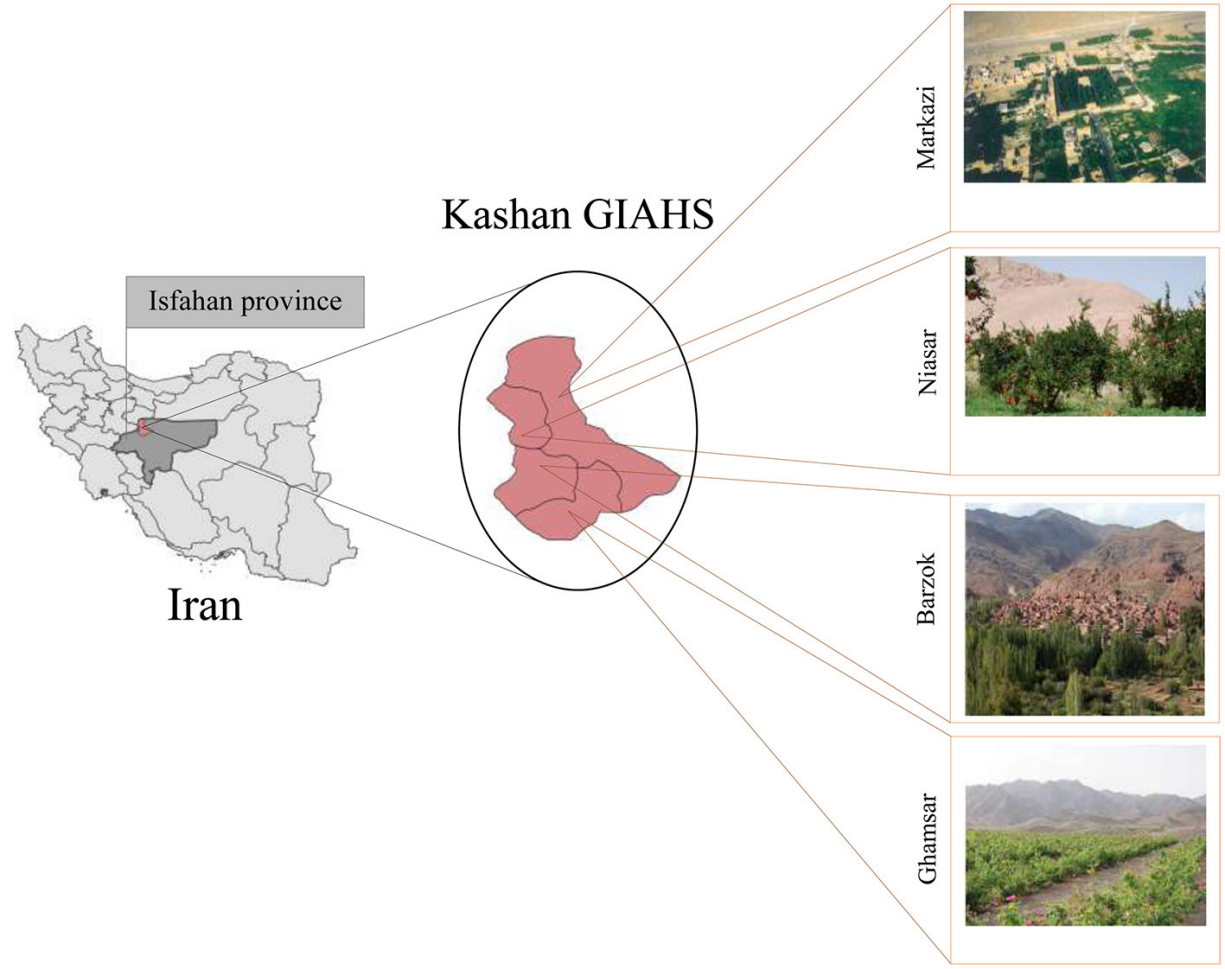
The location of Kashan GIAHS.

Kashan GIAHS as the study area in this study is located in the north-south corridor of Iran and on the way from Tehran to Isfahan and part of the southern cities of the country. Traffic in the area has increased, which has also increased the problems associated with controlling the outbreak of corona in the area. Kashan GIAHS is located in a large area and has 65 villages that have been divided into 4 parts in terms of country divisions. Figure 3 shows the location of Kashan GIAHS.

“Qanat Irrigated Agricultural Heritage Systems, Kashan” is located in Isfahan province and the hot and dry region of Iran. This region is one of the oldest human habitats in Iran and is known as one of the roots of qanat-based agriculture in the world. The selection of agricultural and horticultural species in this region is based on water use efficiency and quality. Indigenous knowledge of qanat management and water distribution in this region is very rich and is a clear example of the ability of the local community to participate in the management of water and soil resources. This management system has various economic, legal, environmental, technical and social dimensions, and its flexibility and dynamism have guaranteed the sustainability of this race for centuries. The dominant agricultural system in the region is family agriculture and livelihood, and related activities in this region are directly and indirectly related to the qanat.

Due to the importance of the qanat in this region, special culture and beliefs have been formed around the qanat, and one of them has been the determination of gender for the qanats. In this arid region, agricultural biodiversity, which is one of the pillars of agricultural heritage, is very rich and no specific product has been introduced as the main product or the most important product. One of the remarkable visual effects in the landscape of Kashan GIAHS is the flower gardens of Mohammadi red rose, which is very famous in the region. Roses and essential oils of these flowers also have significant sales in domestic and foreign markets. The attractiveness of these flower gardens is such that during the tourist season and during the relevant festivals, many tourists visit the flower gardens of Ghamsar region. In GIAHS Kashan, other agricultural systems that are not based on irrigation with qanats are also mentioned, of which the “Chale Sombak” is a clear example. In this arid area, farmers dig holes in the sand dunes to approach groundwater and use soil moisture to grow various crops, especially watermelon (25). In Kashan GIAHS, geographically, three areas can be considered desert, foothill and mountainous, the highest villages are located at an altitude of 2,500 m and the lower villages are located at an altitude of 1,000 m above sea level. Altitude difference is one of the factors in the formation of agricultural biodiversity and is one of the best criteria for biodiversity zoning (33). This feature of altitude difference has caused more enrichment of agricultural biodiversity in Kashan in a relatively small area.

It is about 75% of the water requirement in Kashan which are supplied from qanats. This signifies the critical role of qanats in the food security of the region even today. In total, about 100,000 tons of field crops are produced in Kashan, in an area of about 7,350 hectares. The total production of fruits in the region amounts to 32,000 tons, in an area of about 7,000 hectares. There are about 20,000 farmers in Kashan, who are linked to qanat directly or indirectly. Besides directly contributing to food security by providing reliable irrigation water, qanat irrigated agricultural heritage system helps to sustain food security through retaining and protecting the resource base and being source of fresh water.

Indigenous and important biodiversity species, high value crops, fruits and trees have developed and survived thanks to Qanat technology: the pomegranates, rose flowers, almonds, plums, walnuts, apricots, vines, pistachios, quince, olives, apples, cherry, figs, sour cherry, saffron, pears, peaches, and date plums. There are about 240 selected plant species recorded from the Kashan region. One of the most important varietal biodiversity cherished in the history is the pomegranate, called the “fruit of paradise,” “the seeds of hope,” whose legends and myths were told and transferred from generation to generation. Last but not least, allowing to growing plants in a dry area has permitted to breed local sheep races and shaping habitats for 25 different species of fish, crabs, aquatic plants, invertebrates, and aquatic insects (25).

### Research Method

**Figure 4:**
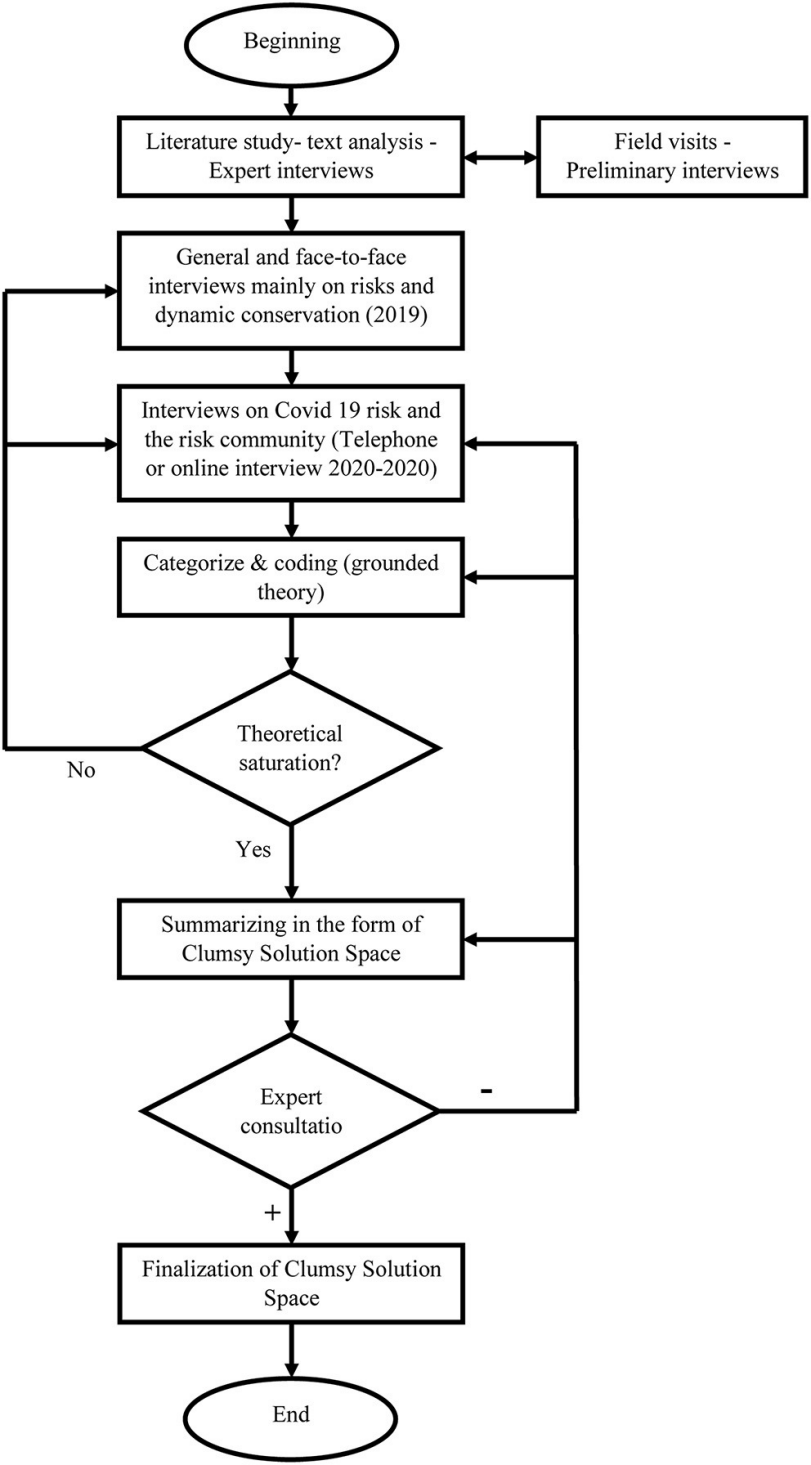
Research steps and procedures.

This research has been done with a qualitative method and the steps taken are shown in Figure 4.

The first two phases of this study were conducted in parallel: the “Studies and Cognition” phase and the “Visits” phase. During 5 trips and field studies from 2018, area identification, field visits, completion of authors' observations and initial interviews were performed and at the same time reviewing the backgrounds related to this research, library records and studies, articles and reports, done. The results of the studies identified some of the issues that should be followed up during the visits, and during the visits, there were encounters with issues that needed to be further studied. Also, in this stage, the initial interviews were conducted in the form of in-depth interviews with 25 people, which provided some of the ideas needed to organize the studies and design the principles of semi-structured interviews in the later stages of the research.

Then, the issues raised in this study, including the risks related to Qanat Irrigated Agricultural Heritage Systems, Kashan, and experiences and ideas of dynamic protection against these risks, were discussed through semi-structured interviews with key informants. The interviews took place in two phases, one phase which was mainly about risks and dynamic protection before the corona pandemic in the first half of 2019 as a face-to-face interview, but the second phase which was mainly related to the corona pandemic and dynamic protection against it. During the years 2020 and 2021, due to the quarantine conditions of Quid 19 and the prohibition of traffic, telephone or video calls were made. It should be noted that the location of Kashan GIAHS in the busy south-north route of Iran caused the prohibition or restriction of traffic during the second phase of this study in this region.

The snowball method was used to understand the complexities of the issue and how to form a dynamic conservation against the existing risks, especially the COVID-19 pandemic, through leading farmers and key informants in the form of semi-structured interviews. Grounded theory was also used in this study as in similar studies (34, 35). The initial interviewees, who were 14 key informants, were searched and introduced in several ways. The channels of introduction of these interviewees were: Kashan city governmental agricultural department, famous qanat and agriculture experts in Iran, in-depth initial interviews conducted in the first phase of the research, review of virtual social networks in the villages of this region and village councils. Finally, 39 key informants from the local community were interviewed to reach the theoretical saturation stage according to the grounded theory. These interviewees covered 34 villages in the region, and in practice all four main regions of Kashan were also covered.

After fully organizing the results of the interviews, the results of content analysis were used to summarize the research and create a space for clumsy solutions. The main output of the research was presented.

## Results and Discussion

### Dimensions of Agricultural Heritage From Different Perspectives

**Figure 5:**
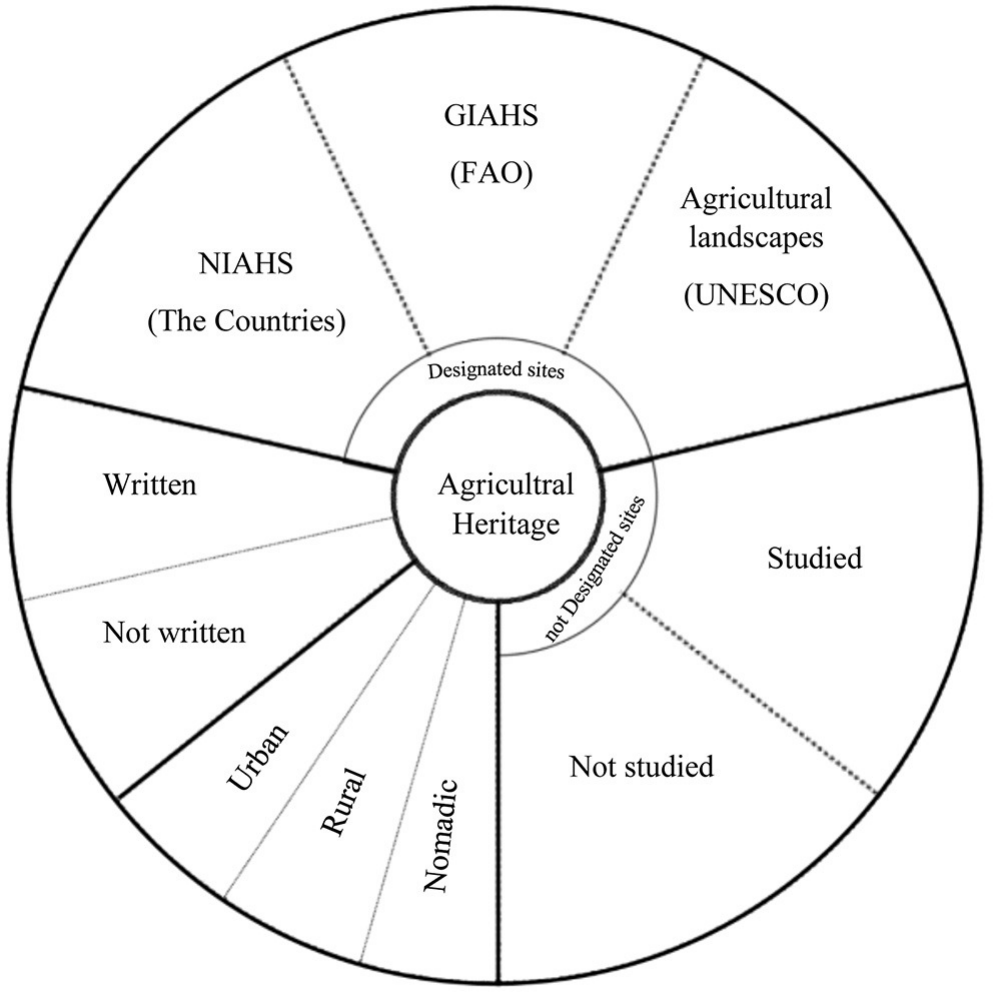
Different dimensions of agricultural heritage.

Based on the first phase of the research, including documentary studies and in-depth interviews with experts, different dimensions of agricultural heritage were identified based on different perspectives. These dimensions are summarized in the Figure 5. This summary has paved the way for a better understanding of the status and roots of Kashan GIAHS for the next stages of research.

In recent decades, with the introduction of the concept of sustainable development in international circles and given the position of small and family farmers in the production of food needed by the people of the world, attention to the importance and necessity of protecting agricultural heritage systems and landscapes has increased. Traditional and family agriculture and with the aim of preserving and improving the functions and values of this agricultural method and in order to achieve the goals of sustainable development, some international organizations focused on the issue of agricultural heritage and in the meantime as shown in Figure 5. The Food and Agriculture Organization of the United Nations (FAO) and the United Nations Educational, Scientific and Cultural Organization (UNESCO), in cooperation with the International Council on Monuments and Sites (ICOMOS), can use various mechanisms to identify, register and dynamically conserve this precious human heritage. From this precious human heritage in Iran, the whole region of Kashan has been registered through GIAHS, and in addition, the Finn qanat garden in Kashan has been registered as one of the Iranian gardens by UNESCO. From another point of view, agricultural heritage is divided into two parts, studied and researched, and not researched. Kashan's agricultural heritage has been researched in the way of registering riots as well as other studies and research on the characteristics of this riots is ongoing. Kashan GIAHS has been studied as an urban and rural area and its urban gardens can be considered as urban agricultural and agro-ecological heritage. Although in Iran and in Persian, numerous sources of agricultural heritage have been published or manuscript sources from the past centuries that have not yet been published, specifically and focused on the Kashan region of written agricultural heritage in this study and research Previous study has not been found, and in fact other Persian books and teachings and skills that are now in the memory of older farmers or passed down from generation to generation can be further studied.

Of course, it should be noted that a very valuable and complete source in 30 chapters about the excavation and management of the qanat in 1010 has been written by the Iranian author Hasb Karaji. The title of this book is “Extraction of Hidden Waters”. Based on the findings of this study and based on interviews with national and local experts, this book, despite addressing the practical details, has not been widely used by people who have been digging and managing the qanat. One reason for this is that the book was written in Arabic, which was the scientific language of Iran at the time.

### Pillars and Relationship of Risk Society, Dynamic Conservation, and Agricultural Heritage

**Figure 6:**
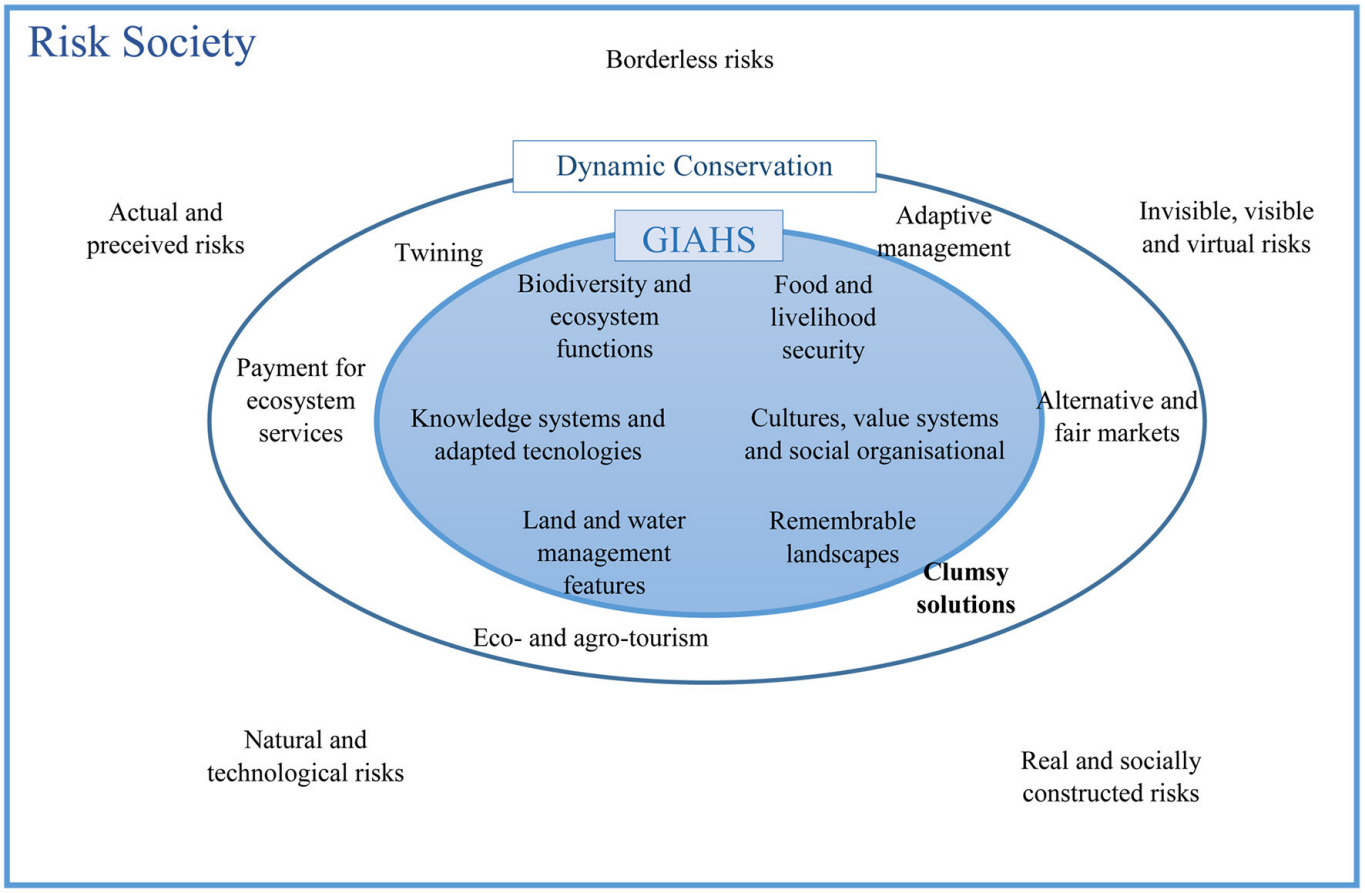
Risk society, dynamic conservation, and agricultural heritage.

In the previous sections, the various sections related to the concepts of risk society, dynamic conservation and GIAHS were explained in Figure 6. Based on the first phase of the research, including documentary studies (22, 36, 37) and in-depth interviews with experts, the appropriateness of this the sections are also summarized together. The new conditions that arise in the risk society have four important characteristics: (1) the sources of support that were used in the past are threatened today; (2) people no longer trust science and expertise; (3) hierarchy and status between lay people and experts are expected to decline; and (4) people are more informed, confident and knowledgeable (38). We consider the problems that arise in such situations as wicked problems. As Ritte and Webber described such problems in 1973, their nature is different at any given time and place, they are dynamic, complex, and multidimensional, they themselves are the result of problems, and they create other problems. And they are not solvable with a particular knowledge, and a set of knowledge and skills is needed to deal with them. In this type of problems, it is not possible to reach a solution by trial and error and generalize it to other situations, because due to the dynamics of these problems, the conditions and results of actions are constantly changing.

The dynamic conservation approach seeks to improve the overall performance of the agricultural heritage system, inspired by nature and based on agroecological processes, indigenous knowledge related to adaptation management and social learning processes, and thus bring greater prosperity to society. It should be noted here that the overall performance of the system should be enhanced without undermining the cultural identity, values of the local community, social relations, agricultural biodiversity and the ecological integrity of the system. This situation can be achieved if there is public awareness, a real and conscious agreement between the local community and other stakeholders and the cultural considerations of the respective communities (9). In this regard, a methodological framework for dynamic protection of agricultural heritage systems has been designed and presented through the FAO, and this framework covers various dimensions of agricultural heritage (9).

Designing dynamic conservation action plans requires an in-depth understanding of the complex social relationships, social impact and structure of local community social institutions. The scope includes several issues including ethnicity, local responsibilities, settlement characteristics, group membership, identity, gender relations, leadership, political institutions; Cultural contexts around the world, such as language, values, rights, knowledge, aesthetics, methods of production, distribution and allocation of labor, include technologies, all of which will have socio-ecological implications for the systems concerned (39, 40).

For dynamic conservation, the use of pre-determined and bottom-up programs will not be useful and it is necessary to formulate a dynamic protection action plan and implement it, from a suitable platform for partnership and formation of communication and dialogue between various stakeholders, including national trustees, National agricultural heritage systems (NIAHS) and customary owners of agricultural heritage systems should be used and in improving this process, educational institutions and non-governmental organizations will also play an effective role (9). Dynamic conservation action plan based on the analysis of threats and challenges in an agricultural system, review of national and local laws and policies affecting the various dimensions of a crop, existing strategies and actions and future plans to be implemented by local and national stakeholders to enhance conservation Dynamically executed, compiled. Therefore, in this study, key informants of the local community have been the main basis for recognizing and developing dynamic conservation measures.

### Clumsy Solution Space

In the face of wicked problems, clumsy solutions are often suggested, which in this study, in addition to other elements of dynamic conservation that have been used in research and other experiments, have been given special attention. The application of these solutions has been increasingly reinforced by empirical evidence (41, 42). They are used for adaptation and development in society (22, 43, 44). A clumsy solution is a set of solutions that combines different perspectives and solutions in a flexible and creative way. The perspectives commonly used in this collection are based on cultural theory and include three different perspectives. Following the egalitarian approach, solutions to wicked problems can be found by promoting a decentralized and self-sustaining society characterized by high empathy and collective intelligence to protect fragile nature. Individualists, on the other hand, address wicked problems by facilitating individual responses such as creative competition and technological innovation. They believe that nature is flexible, while the hierarchy sees the need for stronger regulations and considers nature controllable (42, 44).

In order to face the various risks that have emerged with the advent of COVID-19, some general paths have been formed around the world, such as greater adaptation to technology, especially effective technologies in telecommuting, online sales, etc. Solutions in the local community of Kashan GIAHS has been formed, which has been identified through semi-structured interviews with key informants, and can be related to Clumsy's solutions for wicked problems.

The most obvious measures that make up Clumsy Solution Space for resilience of Kashan qanat-based agricultural heritage are presented in this section. It should be noted that among the 39 key informants interviewed, 7 were over 70 years old who had relatively higher experience, 26 were between 40 and 70 years old and 6 were under 40 years old. Most were interested in gaining experience in newer topics such as growing new crops, domestication wild plants and developing ecotourism.

**Figure 7:**
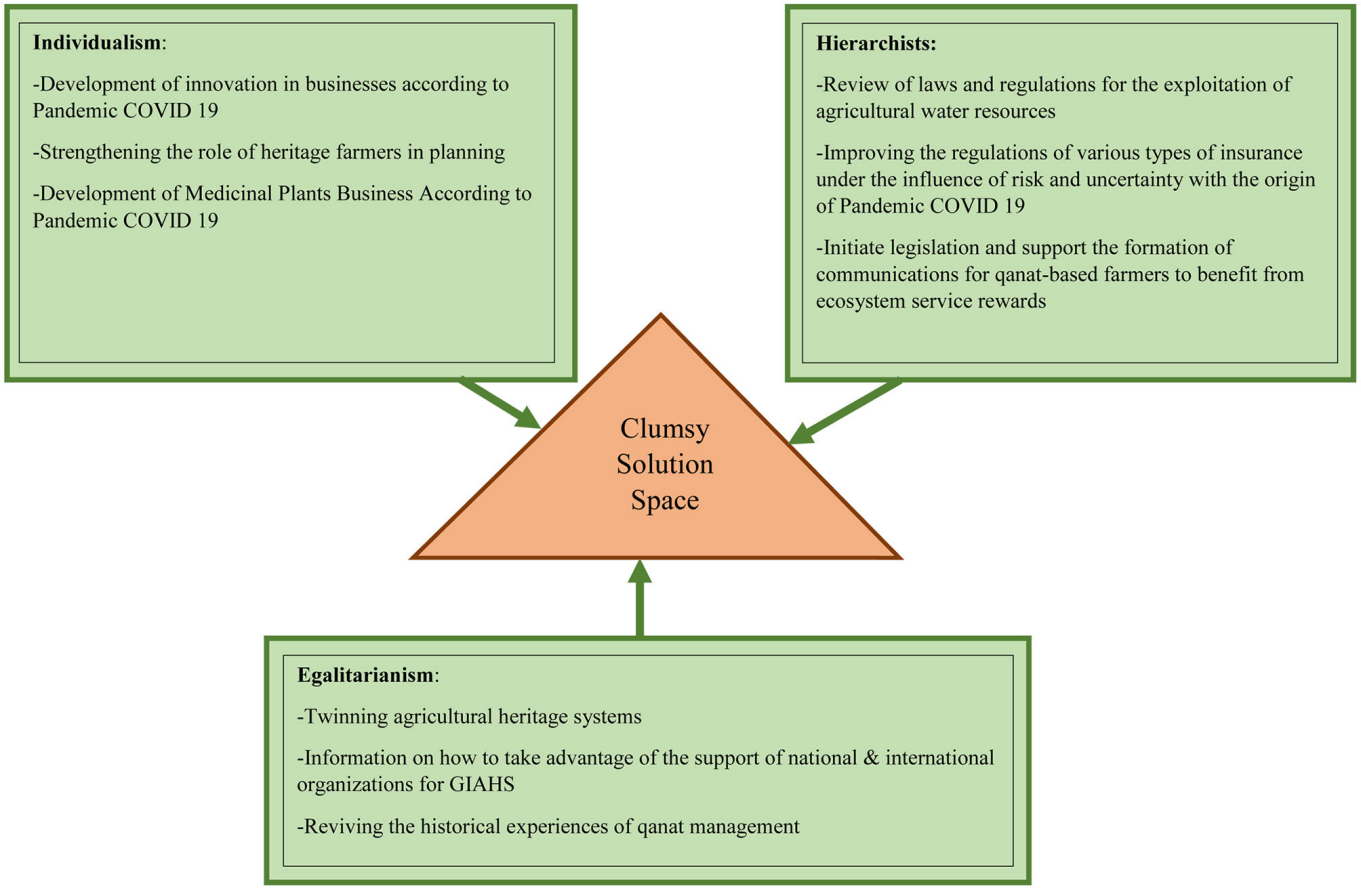
Clumsy solutions space.

During this research, basic information based on literature review, authors' observations and semi-structured interviews with key informants that have been mentioned so far have been obtained. This information was then organized based on grounded theory and content analysis was performed. Solutions related to dynamic conservation through cultural theory were then summarized in three perspectives, and their combination formed the space for Clumsy solutions. The general results of the documentary studies, the opinions of the interviewees and the analyzes performed can be seen in Figure 7, and the relevant explanations are provided below. In the explanation section, considering that the main source of each solution was the interviews conducted, the general and common theme among some of the interviewees is mentioned. Given that a number is assigned to each interviewee, the number of the relevant interviewees is also included.

### Egalitarianism

- Twinning agricultural heritage systems: As experienced in GIAHS (9), Twinning through the development of cooperation and friendship can help the two sister communities in the face of problems such as the risks of COVID-19 and finding solutions Help.

Interviewees Nos. 5, 2, and 24: We have no information or connection with other GIAHS sites in the world, contact with them may lead to the formation of new ideas for us and if our circumstances are similar, we may be able to exchange experiences and etc.

Although none of the interviewees when referring to the problems of the region made any direct reference to the need to establish Twinning with other GIAHS sites or agricultural heritage areas, the solution to the problems they stated in various sources such as Twinning, it has been suggested (45, 46). The use of “Twinning” capabilities is one of the factors that can strengthen the dynamic conservation in a GIAHS area. In fact, twinning expresses the unity and identity that is created by different local communities and is a very flexible platform and the most interesting type of partnership that can be formed between small communities, villages, cities and towns, etc. This capacity also enables the younger generation to connect with their counterparts in different countries, thereby increasing their self-confidence and ability.

- Information on how to take advantage of the support of national and international organizations for GIAHS: Accurate and practical information to farmers about the potential of international institutions to support heritage agriculture and the experiences of other GIAHS in this regard, the conditions for further resilience Prepares in the face of risks.

Interviewees 33, 29, and 20: We do not have any information to be able to use global support and facilities. How did the rest of the GIAHS sites do this? Do the laws of the country allow this?

Farmers and local associations do not have much information about the structure and conditions of various types of support and funds that can directly or indirectly strengthen and protect heritage systems. In some cases, based on misconceptions in this regard, they do not take any action to benefit from the help and support. Also, most do not know where to find information. Based on the experiences gained in different GIAHS areas, subsidies, supports and funds can play a significant role in the formation of dynamic conservation and risk management (11, 12, 47). It should be based on research and inquiries from national and international authorities and should be fully up-to-date, practical and usable. Improve the level of capability and capacity building in Kashan GIAHS.

Reviving the historical experiences of qanat management: Applying the genius and ability of the local community inspired by the historical experiences of participatory agricultural management based on qanats can continue the experience of historical stability of this GIAHS.

Interviewees Nos. 1, 17, 20, and 39: The experiences of our fathers and old farmers and builders and managers of qanats. If not recorded, these experiences may soon be out of reach, but if these experiences as well as old agricultural species are available to us and generations Next, so this heritage can be preserved.

Kashan GIAHS qanats have been preserved throughout history based on local knowledge and the participation and genius of local people (48). This protection could not have been sustainable for hundreds of years if it had not been accompanied by dynamism. Although the formation of a “risk community” in recent decades has damaged the various structures of Kashan GIAHS, but according to the answers of local interviewees and experts, creating and strengthening structures to use genius and collective ability inspired by historical experiences in this region can Make this GIAHS more stable. The experiences of qanats diggers have been recorded to some extent (24) but its agricultural dimensions as well as conservation of heritage seeds and seedlings and agricultural biodiversity of this region have been almost neglected.

### Hierarchy

- Review of laws and regulations for the exploitation of agricultural water resources: Review of laws should be done with more serious attention to the achievements and historical sustainability of qanat agriculture and not only under the influence of knowledge and experience that originates from abroad. In these laws, the regulations for drilling wells and constructing dams in the area of qanats should also be reviewed and tightened.

Interviewees Nos. 6, 11, and 15: If we had dug wells in qanat areas according to what the engineers recommended and the law allowed us to do, our qanats would have dried up as they have dried up in other areas. These laws cannot protect the qanats.

During the past centuries, Qantas has been one of the most important sources of water supply in the Iranian plateau (31) but after considerable application of water pump technology in Iran and also the prevalence of deep wells. Over time, regulations and laws also facilitated the use of wells. These events coincided with the implementation of Principle Four (1949–1971) of the then President of the United States Truman (49). In many cases, this issue led to the formation of risks and sometimes irreparable damage to the qanats. Even in cases such as the village of Shadian, which is one of the desert villages of GIAHS in Kashan, the local community has been completely distrustful of the experts and the issuance of well drilling permits in accordance with the law. Interestingly, they believe that the availability of fresh water and the better condition of their qanat compared to the surrounding villages is due to this resistance to drilling wells. However, some of the desert villages in this GIAHS have lost their freshwater qanats or have been severely damaged, while most of the wells that were drilled in the same villages a few decades ago as the water became saltier over time, they also needed to increase the depth. The salinization of well water over time also makes the soil of the region saltier, and this is exactly the opposite of the process created by freshwater qanats in this area (50). The combination of these factors has led to a relative distrust of regulations and expertise related to drilling wells and the use of water resources in the region, which indicates the need to pay attention to this issue.

Improving the regulations of various types of insurance under the influence of risk and uncertainty with the origin of Pandemic COVID-19: Increasing uncertainty and risk in businesses such as agriculture, agricultural tourism, ecotourism, carpet weaving can be partially compensated by developing different types of insurance.

Interviewees Nos. 4 and 35: Insurance does not provide adequate coverage for the problems that exist in our area during the Corona, and especially for jobs that are sometimes done at home (such as carpet weaving).

Insurance can increase resilience in agricultural communities (51, 52). Insurance for Iran has been one of the services that has become more popular in the country in recent decades and may have been less popular in more traditional areas. corona risks highlighted the need to address this issue in areas such as agriculture, agricultural tourism, nature tourism, carpet weaving, etc. Turns to carpet weaving and other household chores increased during the Corona, but such occupations encountered difficulties in obtaining insurance, which is common for conventional occupations. It is noteworthy that, Kashan handmade carpets (ghali) were historically valuable and had a national and world reputation (53). Also, according to the findings of this study, the income from the sale of carpets as a supplementary income in the years of qanats water shortage and also at a time when events such as floods caused damage to qanat and repairing qanat required a lot of money, plays a very important role. GIAHS has been an important factor in strengthening resilience in the agricultural community based on Kashan GIAHS. Therefore, supporting this profession is necessary for the formation of dynamic conservation in Kashan GIAHS, and this necessity has become more apparent in the Corona period.

- Initiate legislation and support the formation of communications for qanat-based farmers to benefit from ecosystem service rewards: In Iran, there are no relationships under which heritage farmers can benefit from their ecosystem service rewards, such rewards can be Assist communities in meeting old and emerging risks.

Interviewees 8, 21, 29, and 38: Many people explain that the qanat prevents the advance of the desert or the desert villages and oases are beautiful, etc., but when the qanats are spent, we are alone and with our own capital and We have to protect the qanats with great difficulty.

qanat, especially in desert ecosystems, increases biodiversity, whether in the form of agricultural biodiversity or wild biodiversity (54). Also, qanats, which usually flow from the mountains, are less saline than the water of wells in desert areas, and over time, the salinity of desert soils decreases, and thus can prevent the expansion of desert boundaries (55). Despite the ecosystem services mentioned as examples, these services are not compensated in Iran for local communities such as Kashan GIAHS farmers, and it seems that in order to solve the complex problems of this region and strengthen dynamic conservation, formulate structures and regulations in this field. It is also effective and important.

### Individualism

- Development of innovation in businesses according to Pandemic COVID-19: Development of innovations based on business and online sales of products and digitization and application of new technologies while meeting the requirements of sustainability, it is necessary. Also, agricultural tourism, rural tourism and ecotourism can be given more attention because they are outdoor tourism and may have lower risks of spreading diseases such as corona disease. Adapting global e-tourism experiences can also boost innovation in this area.

Interviewees Nos. 14, 26, and 36: If it were not for all kinds of online and offline sales, we would have suffered heavy losses during the Corona or we would have had to sell part of our land. If tourism in our gardens and orchards is properly advertised and well managed, we may be able to sell our products to tourists again after the corona or when the corona is less common.

Before the start of the COVID-19 pandemic, innovations had begun, especially in the methods of selling the product in Kashan. For example, some farmers have welcomed such sales based on suggestions from online sales sites for certain agricultural products. Such innovations became more effective during the COVID-19 pandemic. There have been valuable experiences in countries such as Japan regarding the effectiveness of tourism programs for dynamic conservation of GIAHS (56). These experiences and frameworks can also be used during the corona pandemic years.

- Strengthening the role of heritage farmers in planning: Considering the historical role of these farmers in maintaining social, economic and environmental sustainability of the region, strengthening the role of leadership for them and creating the ground for informal-planning, it is recommended.

Interviewees Nos. 18 and 33: No one pays attention to our opinions, the rules and programs are only announced to us.

It seems that the administrative structures that decide on agricultural frameworks and programs do not pay much attention to the opinions and experiences of farmers. Meanwhile, farmers in Kashan and other GIAHS areas throughout history have succeeded in creating and maintaining impressive and sustainable agricultural systems with global value. Therefore, at least in such areas, more attention can be paid to the advisory role of leading farmers for policy-making.

- Development of Medicinal Plants Business According to Pandemic COVID-19: Kashan GIAHS, based on its rich agricultural biodiversity, has a high potential for the production of medicinal plants as well as the collection of natural and wild medicinal plants. The domestication of wild plants with qanat water, which has similarities to the quality of spring water, has also been considered profitable in the COVID-19 pandemic and can be developed. Due to the prevalence of traditional medicine in large cities close to this GIAHS, more people can engage in this type of business.

Interviewees 5, 7, 25, and 34: Many of the medicinal plants that we grow or are wild in our area, wild plants such as “Dracocephalum kotschyi Boiss” (zarringiah) are good for boosting the body's immunity, and the sale of our products during the corona is very It has become more.

Most of the time, farmers who cultivate medicinal plants are younger, and turning to such species, which are economically valuable and sometimes scarce, can lead to the dynamism of the region's agricultural system and the formation of dynamic conservation. It is also interesting to note that according to interviews with farmers, in some cases the income from 1 year of cultivation of medicinal plants is more than 10 times the income of species that are normally cultivated in Kashan.

## Conclusion

Although rural communities, a significant number of which are located in developing countries and in GIAHS areas, have had successful experiences in sustainable development and dynamic conservation of their heritage, today they face severe shocks that pose community risks. Such shocks have created wicked problems. One of the newest and most obvious of these shocks is the shocks associated with the COVID-19 pandemic. It is important to study the behavior and adaptation of these communities to these shocks and provide solutions in this regard. In this study, an attempt was made to achieve such solutions.

The COVID-19 pandemic further highlighted the power and diversity of risks and the application of risk society theory. Such risks can threaten the various pillars of human civilization, including the valuable heritage that humans have provided in agriculture over thousands of years. In this study, while formulating the various dimensions of agricultural heritage, the theory of risk society was used to create dynamic conservation of qanat Irrigated Agricultural Heritage Systems in Kashan. For this purpose, in addition to documentary studies, field visits and in-depth interviews with experts, key informants of the local community were interviewed. Different stages of research eventually led to the formation of Clumsy Solution Space.

Based on the findings of this study, strengthening the online sales of various rural products of Kashan, especially medicinal plants, as well as rural and community-based tourism can increase its resistance to various shocks by increasing the economic prosperity of the region, especially if in defining regional brands Introduction of identity and characteristics of GIAHS should also be considered. Also, strengthening relationships with other institutions and communities at the national and international levels with examples such as twinning can be a window to share experiences and resources and be effective in adapting to the difficulties of the risk society.

Following some managerial suggestions can also lead to the realization of some clumsy solutions. These include reviewing the rules for exploiting water resources and protecting qanats, strengthening home-based business insurance, defining ecosystem rewards for farmers in rural villages in Kashan, who provide ecosystem services, and governing to facilitate international funds and assistance. The findings of this study can be used in developing an action plan for dynamic conservation of Kashan GIAHS and be inspiring for other regions as well.

This Clumsy Solution Space can play an important role in sharing and developing the experience and views of different sections of the local community of Kashan GIAHS to face the COVID-19 pandemic. Also, these solutions, perspectives and ways to reach them can inspire other communities with agricultural heritage to protect this valuable human heritage. Many areas of Iran that have valuable agricultural heritage have qanat-based agriculture, and such areas exist in other parts of the world. Experiences such as exploiting qanat -based tourism can be similar in other rural areas. Localized and applied. Of course, not all solutions are related to the qanat, and issues such as strengthening the medicinal plant business in the Corona pandemic in other rural communities, especially in areas where potential customers of traditional medicine products are found, can also be adapted and exchanged experiences.

Given that this study deals with the qualitative dimensions of risks, complex problems and related solutions, for future research, it is suggested that quantitative risk assessment research be defined in GIAHS areas and based on risk prioritization and related solutions, define the importance of different solutions in the dynamic conservation action plan of each GIAHS.

## Data Availability Statement

The raw data supporting the conclusions of this article will be made available by the authors, without undue reservation.

## Ethics Statement

Ethical review and approval were not required for the study on human participants in accordance with the local legislation and institutional requirements. Written informed consent for participation was not required for this study in accordance with the national legislation and the institutional requirements.

## Author Contributions

All authors listed have made a substantial, direct, and intellectual contribution to the work and approved it for publication.

## Conflict of Interest

The authors declare that the research was conducted in the absence of any commercial or financial relationships that could be construed as a potential conflict of interest. The reviewer AD declared a shared affiliation with all of the authors to the handling editor at time of review.

## Publisher's Note

All claims expressed in this article are solely those of the authors and do not necessarily represent those of their affiliated organizations, or those of the publisher, the editors and the reviewers. Any product that may be evaluated in this article, or claim that may be made by its manufacturer, is not guaranteed or endorsed by the publisher.
